# Recovery-oriented practices as a pathway toward advancing mental healthcare in Brazil

**DOI:** 10.1590/0102-311XEN134125

**Published:** 2026-04-27

**Authors:** Laís Mendonça de Souza, Tiago Ribeiro da Silva, Marianne Farkas, Zlatka Russinova, Mário César Rezende Andrade

**Affiliations:** 1 Universidade Federal de São João del-Rei, São João del-Rei, Brasil.; 2 Universidade Federal de São Paulo, São Paulo, Brasil.; 3 Boston University, Boston, U.S.A.

## Introduction

Recovery is multifaceted - a personal journey, sociopolitical movement, and guiding orientation -that profoundly shapes global mental healthcare. In Brazil, *Law n. 15,126/2025* ^1^ recently established humanized care as a fundamental principle of its Brazilian Unified National Health System (SUS, acronym in Portuguese). This article defends recovery-oriented practices as a pathway for such care, providing ideas to support the implementation of this law and advance mental healthcare in Brazil.

## The recovery orientation in mental health

Personal recovery originated in the 1970s U.S. psychiatric survivor movement, championing autonomy and meaningful lives beyond illness. First-person narratives have challenged psychiatric dominance and emphasized agency ^2^ ^,^ ^3^ ^,^ ^4^ ^,^ ^5^. Then, 1980s research countered narratives of decline in chronic illness, especially schizophrenia. Longitudinal studies have shown significant symptom and functional improvements, defying pessimistic clinical practice ^6^.

The concept of recovery emerged from lived experiences and empirical research that deemed it as a personal, non-linear process independent of symptom remission. It prioritizes living a meaningful, hopeful, and contributory life despite ongoing symptoms. Recovery involves transforming attitudes, values, goals, and social roles, shifting from “recovery from” to “recovery in” mental illness ^2^ ^,^ ^5^.

Recovery has been traditionally defined as a transformative, individualized journey occurring with and without professional care ^5^. A systematic review integrated these elements into the CHIME framework: Connectedness, Hope, Identity, Meaning, and Empowerment ^7^. This widely used framework helped underpin the development of new recovery-oriented interventions that actively target these domains (as detailed in the following section). Other processes include dealing with difficulties, personal choice, and confronting challenges ^8^.

Knowledge from lived experiences advanced services, fostering interdisciplinary clinical approaches. This perspective acts as an ethical foundation, centering users’ voice as a counterpoint to biomedical models. Widely accepted for humanizing care and framing psychosocial research, this notion has guided policies in pioneering countries such as Australia ^9^ and the United Kingdom ^10^, which adopted Recovery as a fundamental principle for service restructuring. The concept is also a World Health Organization Action Plan goal ^11^ and a guiding principle for global community-based services, aligning itself with person-centered rights-based approaches ^12^.

## Recovery-oriented mental health practices

While recovery can occur independently of psychiatric treatment, mental healthcare providers and services can be crucial facilitators ^13^. A review has found four key domains for professional involvement: supportive relationships, individualized care, organizational commitment, and citizenship/rights ^14^. The recognition of users’ role as citizens, as championed by Rowe ^15^, is crucial, shifting the focus from pathology to social participation and reinforcing self-determination and social belonging − pillars of recovery.

In recent decades, various interventions have promoted recovery. Peer support stands out in recovery-oriented interventions, with individuals with lived experiences assisting others in their own recovery journey ^16^. Numerous reviews have shown its efficacy in improving clinical and psychosocial outcomes, including hope and empowerment. Health systems like the UK National Health Service have formally integrated peer support workers into mental service teams, using them to co-produce care plans and act as models of hope and possibility ^17^.

Other empirically validated recovery-oriented interventions include:

- Self-management groups and programs (e.g., Wellness Recovery Action Planning; Illness Management and Recovery; and Autonomous Medication Management - GAM, acronym in Portuguese), offering structured tools for users to become active agents in managing their health;
- Shared decision-making, ensuring treatment choices are consensual and respecting autonomy;
- Person-centered crisis interventions (e.g., crisis joint plan and open dialogue), which prioritize the social network and immediate intervention, avoiding coercion and unnecessary hospitalization;
- Individual placement and support for employment, a place-and-train model proving significantly more effective than traditional vocational models;
- Recovery colleges, educational settings offering co-produced courses on recovery and self-management;
- Recovery-oriented cognitive therapy and positive psychotherapy for psychosis ^16^ ^,^ ^18^.

These interventions often work via four mechanisms: providing information/skill development, establishing therapeutic alliances, offering role models, and enhancing choice/opportunity ^19^. They are crucially influenced by clinician attitudes. Professionals must broaden their perspectives, fostering hope, tolerance for uncertainty, and belief in the possibility of recovery ^13^ ^,^ ^20^.

Advancing mental healthcare in Brazil: aligning care with recovery-oriented practices

The Brazilian psychiatric deinstitutionalization, driven by social movements, transitioned mental healthcare from institutions to community-based services, prioritizing human rights and social inclusion ^21^ ^,^ ^22^. While Brazil’s mental health policy fails to explicitly adopt recovery orientation, its principles are highly compatible with it, suggesting avenues for advancement.

The reform suffered the influence of international deinstitutionalization, notably the Italian model, which championed psychosocial rehabilitation and community integration ^21^ ^,^ ^22^. Also, the Brazilian pursuit of universal access and human rights elements shaped its public health policies, establishing the SUS. Enshrined in the 1988 *Brazilian Federal Constitution*, SUS guarantees health access and human rights, emphasizing intersectoral action and social participation based on three principles: comprehensiveness, universality, and equity ^23^. Recently, *Law n. 15,126/2025* formally established humanized care as an additional fundamental SUS principle ^1^. Humanization had been integral to SUS via the National Humanization Policy (HumanizaSUS) for two decades ^24^. This enactment underscores the timeliness of discussing recovery-oriented practices as a pathway to expand current practices and ensure the implementation of that law.

The connection between humanized care (*Law n. 15,126/2025*) and recovery lies in the human rights framework, notably the United Nations Convention on the Rights of Persons with Disabilities (UNCRPD) ^25^ and High Commissioner reports ^26^. SUS humanization demands respect for autonomy, will, and preferences. Recovery acts as the operational orientation enacting these rights. While the law mandates humanization, recovery provides interventions (e.g., peer support, shared decision-making), ensuring person-centered non-coercive care that promotes citizenship.

Based on human rights defense via the *Caracas Declaration* ^27^, Brazilian psychiatric deinstitutionalization guided the development of the Psychosocial Care Network (RAPS, acronym in Portuguese) and Psychosocial Care Centers (CAPS, acronym in Portuguese), the country’s main community mental health services. CAPS use interdisciplinary practices, focusing on support and welcoming. The Brazilian Singular Therapeutic Plan (PTS, acronym in Portuguese) configures a key collaborative strategy for person-centered planning. The Brazilian policy also promotes intersectoral strategies to enhance social inclusion, income generation, and employment for individuals with mental disorders ^24^.

The literature evinces a growing consensus in the underlying principles of the Brazilian model − humanization, social control, human rights, and person-centered care − form crucial foundations in line with and advancing recovery-oriented approaches ^4^ ^,^ ^21^ ^,^ ^22^ ^,^ ^28^ ^,^ ^29^ ^,^ ^30^. For instance, SUS humanization practices such as PTS and the expanded clinic can be enriched by recovery ideas (e.g., focus on hope, citizenship, non-coercion, and empowerment). These ideas can transform the PTS from a clinical document into a robust life plan centered on users’ personal aspirations and guide the expanded clinic toward mobilizing personal and social resources, promoting full inclusion (citizenship domains, as per Rowe ^15^).

Despite recent progress in Brazil, practical implementation faces numerous challenges. Many professionals are still oriented toward a disease-centered model, characterized by hierarchical relationships with users ^21^ ^,^ ^22^. Service indicators often rely on clinical parameters only focused on symptom remission and adherence ^22^ ^,^ ^24^.

A key challenge is ensuring adequate service coverage and model implementation across the vast Brazilian territory. This demands greater investment in equitable CAPS distribution, coordinated primary care (Family Health Strategy), and the continuous hiring and training of professional teams ^22^ ^,^ ^31^. These efforts are crucial to preserve quality and align efforts with guiding principles.

Advancing toward recovery also requires shifting from professional-centered care to collaborative relationships that promote empowerment and self-management based on users’ choices. Increasing quality-of-care management practices such as PTS is an example ^21^ ^,^ ^28^. Evidence suggests expanding training programs in recovery-oriented practices for healthcare providers can effectively implement this orientation, as in international examples ^19^.

A crucial step involves including peer specialists to coordinate peer support groups and enhance user engagement practices ^4^ ^,^ ^13^ ^,^ ^19^. Reinforcing the pivotal role of individuals with lived experiences, the *Final Report of the 5th National Mental Health Conference* explicitly mentions recovery and defends the adoption of peer support to strengthen national care ^30^ ^,^ ^32^. This reflects a growing consensus among researchers and activists that peer support is a key pathway to integrate recovery-oriented practices in Brazil and value experiential knowledge ^4^ ^,^ ^30^ ^,^ ^33^. Examples include the strong national network of Hearing Voices groups and university/NGO projects ^30^ ^,^ ^33^. Despite these efforts and pilot initiatives, such as harm reduction workers with lived experiences in Belo Horizonte (Minas Gerais State) ^33^ and the adaptation and implementation of peer support in Campinas (São Paulo State) ^34^, the formal integration of peer workers into service teams is yet to configure a national policy.

Also, GAM groups are increasingly implemented nationwide. Developed by the Quebec (Canada) user movement to foster empowerment, they are run by professionals or peers ^35^. Beyond these advancements, innovative models such as the finnish open dialogue for crisis management have also been introduced, despite no official implementation ^36^.

An additional viable path involves empowering users, families, and communities by educational services such as recovery colleges ^16^ (found globally but still incipient in Latin America). Their principles align themselves well with popular health education and Paulo Freire’s framework ^37^. While UK Recovery Colleges have faced criticism regarding their institutionalization, their core principles (co-production, non-clinical focus, and hope and empowerment promotion) remain highly valuable. Their adaptation to Brazil must be rigorously aligned with social control and anti-asylum principles to prevent co-optation.

Strengthening SUS social control mechanisms could also advance strategies promoting citizenship and empowerment, boosting participation in society. Increased investment in community centers, income generation, and continuous anti-stigma initiatives involving society would be vital. In conclusion, this manuscript offers an initial discussion on recovery orientation as a potential possibility to advance mental healthcare in Brazil, especially by valuing experiential knowledge and the leading role of people with lived experiences, agreeing with and contributing to the established principles of the Brazilian Psychiatric Reform and SUS. However, detailing how this orientation can assist in this advancement in convergence with the obtained advances require further and deeper discussions.
