# Supplementary material for: Recovery-oriented practices as a pathway toward advancing mental healthcare in Brazil
Source: Cad Saude Publica. 2026 Apr 27;42:e00134125. doi: 10.1590/0102-311XEN134125 (PMC13170108; doi:10.1590/0102-311XEN134125)
Supplement: Translation

# Translation: Práticas orientadas ao *recovery* como caminho para o avanço do cuidado à saúde mental no Brasil

**DOI:** 10.1590/0102-311XPT134125

**Authors:** Laís Mendonça de Souza, Tiago Ribeiro da Silva, Marianne Farkas, Zlatka Russinova, Mário César Rezende Andrade

**Affiliations:** 1 Universidade Federal de São João del-Rei, São João del-Rei, Brasil.; 2 Universidade Federal de São Paulo, São Paulo, Brasil.; 3 Boston University, Boston, U.S.A.

## Introdução

O *recovery* é multifacetado - uma jornada pessoal, um movimento sociopolítico e uma orientação norteadora - e tem moldado profundamente a abordagem da saúde mental globalmente. No Brasil, a *Lei nº 15.126/2025* estabeleceu recentemente o cuidado humanizado como princípio fundamental do Sistema Único de Saúde (SUS) ^1^. Este artigo defende as práticas orientadas ao *recovery* como um caminho para a implementação desse cuidado, apresentando propostas que podem apoiar a implementação da lei e o avanço da atenção à saúde mental no país. [Optou-se por manter o termo original em inglês (*recovery*) - em alinhamento com documentos oficiais da Organização Mundial da Saúde (OMS) e da Organização Pan-Americana da Saúde (OPAS) disponibilizados em português - por designar um processo multidimensional de empoderamento e cidadania que ultrapassa a mera recuperação clínica e não possui tradução equivalente em português].

## A orientação ao *recovery* em saúde mental

O conceito de *recovery* em saúde mental teve origem no Movimento de Sobreviventes da Psiquiatria (*Psychiatric Survivors Movement*), nos Estados Unidos, na década de 1970, o qual defendia autonomia e vidas significativas para além do transtorno mental. Narrativas em primeira pessoa desafiaram a hegemonia psiquiátrica, enfatizando o protagonismo dos próprios sujeitos ^2^ ^,^ ^3^ ^,^ ^4^ ^,^ ^5^. Paralelamente, pesquisas realizadas na década de 1980 contestaram narrativas de declínio associadas aos transtornos mentais crônicos, especialmente à esquizofrenia. Estudos longitudinais demonstraram melhorias significativas nos sintomas e no funcionamento, contrariando o pessimismo presente na prática clínica ^6^.

O conceito de *recovery* emergiu tanto das experiências vividas quanto da pesquisa empírica, reconhecendo-o como um processo pessoal, não linear e independente da remissão total dos sintomas. Esse conceito prioriza viver uma vida significativa, com esperança e contribuição social apesar da presença de sintomas. *Recovery* envolve transformações em atitudes, valores, metas e papéis sociais, deslocando o foco de “*recovery* da” para “*recovery* na” condição mental ^2^ ^,^ ^5^.

Tradicionalmente, *recovery* é definido como uma jornada transformadora e individualizada, que pode ocorrer com ou sem cuidado profissional ^5^. Uma revisão sistemática integrou esses elementos no modelo teórico CHIME (acrônimo em inglês): Conexão (*Connectedness*), Esperança (*Hope*), Identidade (*Identity*), Sentido (*Meaning*) e Empoderamento (*Empowerment*) ^7^. Esse modelo amplamente utilizado fundamentou o desenvolvimento de novas intervenções orientadas ao *recovery* que atuam diretamente nesses domínios, conforme detalhado a seguir. Outros processos incluem lidar com dificuldades, exercer escolhas pessoais e enfrentar desafios ^8^.

O conhecimento oriundo das experiências vividas contribuiu para o aprimoramento dos serviços, promovendo abordagens clínicas interdisciplinares. Essa perspectiva funciona como base ética, colocando a voz do usuário no centro, em contraponto aos modelos biomédicos tradicionais. Amplamente reconhecida por humanizar o cuidado e orientar pesquisas psicossociais, essa abordagem fundamentou políticas em países pioneiros como Austrália ^9^ e Reino Unido ^10^, os quais adotaram o *recovery* como princípio estruturante da reorganização dos serviços. O conceito também integra o Plano de Ação da Organização Mundial da Saúde ^11^ e constitui princípio orientador de serviços comunitários em todo o mundo, alinhando-se a abordagens centradas na pessoa e baseadas em direitos humanos ^12^.

## Práticas em saúde mental orientadas ao recovery

Embora o *recovery* possa ocorrer independentemente do tratamento psiquiátrico, profissionais e serviços de saúde mental podem ser facilitadores cruciais ^13^. Uma revisão identificou quatro domínios-chave para o envolvimento profissional: relações de apoio, cuidado individualizado, compromisso organizacional e cidadania/direitos ^14^. O reconhecimento do usuário como cidadão, conforme defendido por Rowe ^15^, é central, deslocando o foco da patologia para a participação social e reforçando autodeterminação e pertencimento - pilares do *recovery*.

Nas últimas décadas, diversas intervenções tem promovido o *recovery*. O suporte de pares destaca-se entre elas, envolvendo pessoas com experiência vivida que auxiliam outras em seus próprios processos de *recovery* ^16^. Revisões demonstram sua eficácia na melhoria de desfechos clínicos e psicossociais, incluindo esperança e empoderamento. Sistemas como o Serviço Nacional de Saúde do Reino Unido tem integrado formalmente especialistas pares às equipes de saúde mental, envolvendo-os na coprodução de planos de cuidado e como modelos de esperança e possibilidade ^17^.

Outras intervenções orientadas ao *recovery*, validadas empiricamente, incluem:

- Grupos e programas de autogestão (por exemplo, *Wellness Recovery Action Planning* [Plano de Ação para Recuperação do Bem-Estar]; *Illness Management and Recovery* [Gestão e Recuperação de Doenças]; e Gestão Autônoma da Medicação [GAM], os quais oferecem ferramentas estruturadas para que usuários se tornem agentes ativos na gestão de sua saúde;
- *Shared decision-making* (tomada de decisão compartilhada), garantindo que escolhas terapêuticas sejam consensuais e respeitem a autonomia;
- Intervenções centradas no indivíduo em situações de crise (por exemplo, planos de crise compartilhados e diálogo aberto), que priorizam a rede social e a intervenção imediata, evitando coerção e hospitalizações desnecessárias;
- Apoio individualizado à inserção no trabalho, caracterizado pela inserção direta no emprego com treinamento em serviço, comprovadamente mais eficaz do que os modelos vocacionais tradicionais;
- *Recovery colleges* - centros educacionais de desenvolvimento e *recovery* em saúde mental -, iniciativa baseada na coprodução que oferece cursos voltados ao *recovery* e à autogestão;
- Terapia cognitiva orientada ao *recovery* e psicoterapia positiva para psicose ^16^ ^,^ ^18^.

Essas intervenções frequentemente operam por meio de quatro mecanismos: oferta de informação e desenvolvimento de habilidades; estabelecimento de aliança terapêutica; disponibilização de modelos de referência; e ampliação das possibilidades de escolha e oportunidades ^19^. Sendo fortemente influenciadas pelas atitudes dos profissionais de saúde mental, estes devem ampliar suas perspectivas, promovendo esperança, tolerância à incerteza e crença na possibilidade de *recovery* ^13^ ^,^ ^20^.

## Avanço da atenção à saúde mental no Brasil: alinhamento com práticas orientadas para o *recovery*

A desinstitucionalização psiquiátrica brasileira, impulsionada por movimentos sociais, promoveu a transição da atenção à saúde mental de instituições para serviços comunitários, priorizando os direitos humanos e a inclusão social ^21^ ^,^ ^22^. Embora a política brasileira de saúde mental não adote explicitamente a orientação ao *recovery*, seus princípios são amplamente compatíveis, indicando caminhos para seu aprimoramento.

A reforma foi influenciada pelos processos internacionais de desinstitucionalização, especialmente pelo modelo italiano com sua ênfase na reabilitação psicossocial e integração comunitária ^21^ ^,^ ^22^. Concomitantemente, a busca brasileira pelo acesso universal e pela incorporação de elementos de direitos humanos moldou suas políticas públicas de saúde, culminando na criação do SUS. Instituído pela *Constituição Federal* de 1988, o SUS garante o direito à saúde e aos direitos humanos, enfatizando a ação intersetorial e a participação social, com base em três princípios: integralidade, universalidade e equidade ^23^. Recentemente, a *Lei nº 15.126/2025* estabeleceu formalmente o cuidado humanizado como princípio fundamental adicional do SUS ^1^. A humanização já era componente estruturante do SUS há duas décadas, por meio da Política Nacional de Humanização (HumanizaSUS) ^24^. A promulgação da lei reforça a pertinência de discutir práticas orientadas para o *recovery* como estratégia para ampliar as práticas atuais e assegurar sua implementação.

A conexão entre o cuidado humanizado (*Lei nº 15.126/2025*) e o *recovery* fundamenta-se nos princípios dos direitos humanos, especialmente na Convenção das Nações Unidas sobre os Direitos das Pessoas com Deficiência (CDPD) ^25^ e nos relatórios do Alto Comissariado das Nações Unidas para os Direitos Humanos ^26^. A humanização no SUS exige respeito à autonomia, à vontade e às preferências. O *recovery* atua como orientação operacional para a concretização desses direitos. Embora a lei determine a humanização, o *recovery* oferece intervenções (como suporte de pares e tomada de decisão compartilhada) que asseguram cuidado centrado na pessoa, não coercitivo e promotor de cidadania.

Com base na defesa dos direitos humanos expressa na *Declaração de Caracas* ^27^, a desinstitucionalização psiquiátrica brasileira orientou o desenvolvimento da Rede de Atenção Psicossocial (RAPS) e dos Centros de Atenção Psicossocial (CAPS), principais serviços comunitários de saúde mental do país. Os CAPS adotam práticas interdisciplinares, com ênfase no acolhimento e no apoio. O Projeto Terapêutico Singular (PTS) constitui uma estratégia colaborativa vital para o planejamento centrado na pessoa. A política brasileira também promove estratégias intersetoriais voltadas à inclusão social, geração de renda e inserção no trabalho para pessoas com transtornos mentais ^24^.

Há consenso crescente na literatura de que os princípios estruturantes do modelo brasileiro - humanização, controle social, direitos humanos e cuidado centrado na pessoa - constituem bases fundamentais que se alinham e podem fortalecer abordagens orientadas ao *recovery* ^4^ ^,^ ^21^ ^,^ ^22^ ^,^ ^28^ ^,^ ^29^ ^,^ ^30^. Por exemplo, práticas de humanização do SUS, como o PTS e a clínica ampliada, podem ser enriquecidas por contribuições do *recovery* (como foco na esperança, cidadania, não coerção e empoderamento). Tais contribuições podem transformar o PTS de um documento clínico em um projeto de vida robusto, centrado nas aspirações pessoais do usuário, e orientar a clínica ampliada para a mobilização de recursos pessoais e sociais, promovendo inclusão plena (nos domínios da cidadania, conforme Rowe ^15^).

Apesar dos avanços recentes no Brasil, a implementação prática enfrenta inúmeros desafios. Muitos profissionais ainda se orientam por um modelo centrado na doença, caracterizado por relações hierárquicas com os usuários ^21^ ^,^ ^22^. Os indicadores de serviço frequentemente se baseiam apenas em parâmetros clínicos, focados na remissão de sintomas e na adesão ao tratamento ^22^ ^,^ ^24^.

Um desafio central é assegurar cobertura adequada dos serviços e implementação do modelo em todo o vasto território brasileiro. Isso exige maior investimento para distribuição equitativa dos CAPS, articulação com a atenção primária (Estratégia Saúde da Família) e contratação e qualificação contínuas das equipes profissionais ^22^ ^,^ ^31^. Tais esforços são fundamentais para preservar a qualidade e garantir alinhamento com os princípios orientadores.

Avançar na direção do *recovery* também requer a transição de um cuidado centrado no profissional para relações colaborativas que promovam empoderamento e autogestão, baseadas nas escolhas do usuário. A qualificação de práticas de gestão do cuidado, como o PTS, é um exemplo ^21^ ^,^ ^28^. Evidências indicam que o desenvolvimento de programas de formação em práticas orientadas ao *recovery* pode contribuir nesse contexto, como demonstrado em experiências internacionais ^19^.

Um passo crucial consiste na inclusão de especialistas pares para coordenar grupos de apoio entre pares e fortalecer práticas de engajamento dos usuários ^4^ ^,^ ^13^ ^,^ ^19^. Reafirmando o papel central das pessoas com experiência vivida, o *Relatório Final da 5ª Conferência Nacional de Saúde Mental* menciona explicitamente o *recovery* e defende a adoção do suporte de pares como estratégia para o fortalecimento do cuidado em âmbito nacional ^30^ ^,^ ^32^. Esse ponto reflete o consenso crescente na literatura de que o suporte de pares constitui uma via fundamental para integrar práticas orientadas para o *recovery* no Brasil, valorizando o conhecimento experiencial ^4^ ^,^ ^30^ ^,^ ^33^. Exemplos incluem a consolidada rede nacional de grupos de Ouvidores de Vozes e projetos vinculados a universidades e organizações da sociedade civil ^30^ ^,^ ^33^. Apesar desses esforços e de iniciativas-piloto - como a atuação de trabalhadores de redução de danos com experiência vivida em Belo Horizonte (Minas Gerais) ^33^ e a adaptação e implementação do apoio entre pares em Campinas (São Paulo) ^34^ - a integração formal de suporte de pares às equipes de serviço ainda não se configura como política nacional.

Ao mesmo tempo, grupos de GAM vêm sendo progressivamente implementados no país. Desenvolvida pelo movimento de usuários do Quebec (Canadá) com o objetivo de promover empoderamento, a GAM pode ser conduzida por profissionais ou por pares ^35^. Além desses avanços, modelos inovadores como o diálogo aberto, originado na Finlândia para o manejo de crises, também foram introduzidos, embora ainda não tenham sido oficialmente implementados ^36^.

Outro caminho promissor envolve o fortalecimento de usuários, familiares e comunidades por meio de dispositivos educacionais como os *recovery colleges* ^16^ (presentes globalmente, mas ainda incipientes na América Latina). Seus princípios dialogam com a educação popular em saúde e com o referencial de Paulo Freire ^37^. Embora os *recovery colleges* no Reino Unido tenham sido alvo de críticas quanto ao risco de institucionalização, seus princípios centrais - coprodução, foco não clínico e promoção de esperança e empoderamento - permanecem altamente relevantes. Sua adaptação ao contexto brasileiro deve estar rigorosamente alinhada ao controle social e aos princípios antimanicomiais, a fim de evitar processos de cooptação.

Fortalecer os mecanismos de controle social do SUS também pode impulsionar estratégias voltadas à promoção da cidadania e do empoderamento, ampliando a participação social. Investimentos em centros comunitários, geração de renda e iniciativas contínuas de enfrentamento do estigma, com envolvimento da sociedade, são igualmente fundamentais. Em síntese, este manuscrito apresenta uma discussão inicial ao propor a orientação ao *recovery* como possibilidade de avanço da atenção à saúde mental no Brasil, especialmente ao valorizar o conhecimento experiencial e o protagonismo de pessoas com experiência vivida, em consonância com os princípios consolidados da Reforma Psiquiátrica brasileira e do SUS. Contudo, são necessárias discussões adicionais e mais aprofundadas para explicitar como essa orientação pode contribuir para tal avanço, em convergência com as conquistas já alcançadas.
